# Response to a letter to the editor

**DOI:** 10.1017/S1368980026102961

**Published:** 2026-07-06

**Authors:** Julie Maree Wood, Alison O. Booth, Rebecca Lindberg, Claire Margerison

**Affiliations:** 1 Institute of Physical Activity and Nutrition, Deakin University, Australia; 2 Deakin University, Australia

**Keywords:** food security, refugee, information resources, nutrition literacy, refugee nutrition

(Original Article – *Australian settlement workers’ use of food security information resources with refugee clients: a qualitative exploration)*

We appreciate the interest that the letter to the editor has shown in response to our article ‘*Australian settlement worker’s use of food security information resources with refugee clients’* ^(1)^. We thank the editor for the opportunity to respond.

We agree with the letter’s assertion that food security is complex and that providing access to resources does not address the issue’s broader structural determinants. Our paper is not advocating for a reliance on resource-based approaches as a primary method for addressing the complex web of issues related to food security for people living as refugees. Indeed, the authors readily acknowledge that access to information resources is just one small part of addressing the myriad, interdependent barriers that can impact all dimensions of food security. The paper’s authors further acknowledge that the structural determinants outlined in the letter, including food affordability and transport, must be addressed if the root causes of food security are to be addressed. To this end, the study from which our paper was drawn also explored the contemporaneous food security issues being experienced by refugee populations resettling in Australia. A subsequent paper was published to present these findings^(2)^.

The second paper from our study reported that key structural determinants, such as inadequate social protection, housing scarcity and food inflation, are well-known priorities for food security^(2)^. However, the paper also discussed the heterogeneity of refugee populations. Too often in the literature, we see refugees referred to as a single group when they are actually from widely different regions, tribes, countries, cultures, and food cultures. These markedly different populations also have different pre-settlement pathways, timelines and experiences, which can influence and impact their food security challenges during resettlement^(2)^. This vast heterogeneity is one of the many reasons why a multi-faceted, nuanced approach is required to support food security for people living as refugees^(3)^. Access to accurate and culturally appropriate information resources is just one small, but important, part of that nuanced approach.

Depending on the pre-settlement journey, access to education and the subsequent literacy levels of some refugee populations may be limited^(4,5)^. As the circumstances and timelines of different populations’ pre-settlement journey are highly varied, other populations do have access to education, from primary to tertiary, and their subsequent literacy levels are high^(2)^. Our study found that for those populations whose pre-settlement pathways had afforded them access to education, access to information resources was perceived as particularly valuable. For others with lower literacy levels, the perceived value of the resources was lower and their resource needs differed^(2)^. The findings reinforce our assertion that access to information resources is important but their value varies between different cultural groups and populations, and the resources do not address the broader, structural determinants of this wicked problem.

It is imperative that this heterogeneity of different populations and cultural groups is acknowledged and considered when food security responses and support mechanisms are developed. This includes providing access to suitable, accurate, and culturally appropriate information resources while addressing the broader structural barriers and determinants of food insecurity for people living as refugees.
